# The largest HIV-1-infected T cell clones in children on long-term combination antiretroviral therapy contain solo LTRs

**DOI:** 10.1128/mbio.01116-23

**Published:** 2023-08-02

**Authors:** Johannes C. Botha, Dimiter Demirov, Carli Gordijn, Mary Grace Katusiime, Michael J. Bale, Xiaolin Wu, Daria Wells, Stephen H. Hughes, Mark F. Cotton, John W. Mellors, Mary F. Kearney, Gert U. van Zyl

**Affiliations:** 1 Stellenbosch University, Cape Town, South Africa; 2 Cancer Research Technology Program, Leidos Biomedical Research, Inc., Frederick National Laboratory for Cancer Research, Frederick, Maryland, USA; 3 HIV Dynamics and Replication Program, National Cancer Institute, Frederick, Maryland, USA; 4 Laboratory of Epigenetics and Immunity, Department of Pathology and Laboratory Medicine, Weill Cornell Medicine, New York, New York, USA; 5 Division of Infectious Diseases, Department of Medicine, University of Pittsburgh School of Medicine, Pittsburgh, Pennsylvania, USA; The University of North Carolina at Chapel Hill School of Medicine, Chapel Hill, North Carolina, USA

**Keywords:** HIV, provirus, solo LTR, proviral quantification, HIV cell clones

## Abstract

Combination antiretroviral therapy (cART) suppresses viral replication but does not cure HIV infection because a reservoir of infectious (intact) HIV proviruses persists in long-lived CD4+T cells. However, a large majority (>95%) of HIV-infected cells that persist on effective cART carry defective (non-infectious) proviruses. Defective proviruses consisting of only a single LTR (solo long terminal repeat) are commonly found as endogenous retroviruses in many animal species, but the frequency of solo-LTR HIV proviruses has not been well defined. Here we show that, in five pediatric donors whose viremia was suppressed on cART for at least 5 years, the proviruses in the nine largest clones of HIV-infected cells were solo LTRs. The sizes of five of these clones were assayed longitudinally by integration site-specific quantitative PCR. Minor waxing and waning of the clones was observed, suggesting that these clones are generally stable over time. Our findings show that solo LTRs comprise a large fraction of the proviruses in infected cell clones that persist in children on long-term cART.

**IMPORTANCE:**

This work highlights that severely deleted HIV-1 proviruses comprise a significant proportion of the proviral landscape and are often overlooked.

## INTRODUCTION

Combination antiretroviral therapy (cART) achieves sustained virologic suppression in most people living with HIV-1 (PLWH) who are adherent to their treatment regimens. Nevertheless, current cART does not cure HIV-1 infection (1). The main barrier to curing HIV-1 infection is a reservoir of long-lived, infected cells carrying intact, infectious proviruses (the integrated DNA form of the virus genome). When individuals initiate cART in chronic infection, cells carrying genetically diverse populations of HIV proviruses persist throughout the body (2 - 4). The persistence during cART of proviruses that have identical sequences suggested that cells infected prior to cART initiation could expand through cellular proliferation (5 - 8). HIV integration site analyses showed that infected cells can expand and that the expanded clones can persist for decades (9, 10). Some of these expanded cell clones carry replication-competent (intact) proviruses and comprise the HIV reservoir that leads to viral rebound if cART is stopped. Cell clones that carry defective or intact proviruses can wax and wane over time (11 - 13), although most are highly stable (14). Some clones of infected cells produce enough virus to be detected in the blood by standard plasma HIV RNA assays despite effective cART (11, 15).

Multiple molecular assays have been developed by various groups to determine the integration sites of HIV proviruses (9, 16 - 20). However, these assays provide little information on the sequences of their linked HIV genomes. Additional analysis is needed to determine the sequences of the proviruses for which the integration sites have been determined (15, 21 - 23). Combining these approaches makes it possible to connect integration sites with the sequences of the linked proviruses. In most cases, such methods use HIV-targeted PCR primers to amplify the proviruses. As many proviruses have large internal deletions (24, 25), these assays selectively amplify genomes that contain the appropriate primer-binding sites. LTRs are duplicated in all intact and many defective proviruses; PCR amplification reactions that rely on the use of an internal HIV primer would not be able to amplify proviruses that have been reduced to a solo LTR. Solo LTRs are not part of the HIV replication-competent reservoir. Nevertheless, they are not an uncommon outcome of retroviral integration, as evident from the high prevalence of solo LTRs among retroviral elements in the mammalian germline (9, 26 - 28). Solo LTRs occur through homologous recombination of the LTRs after integration (28 - 30) and have accumulated in endogenous retroviruses over thousands of years. A report of an HIV solo-LTR provirus in a large cell clone from one adult donor suggests that HIV can undergo similar recombination events that lead to the emergence of solo LTRs, like their retroviral ancestors (31). It is not clear how prevalent solo LTRs are in HIV infections, although the ratio of LTR to internal HIV sequences reported by Anderson et al. (31) suggests that they may be common.

Here, we apply an integration site-specific proviral amplification approach to characterize HIV proviruses in clones of cells that persisted in children on long-term cART (>7 years), and show that large, infected cell clones frequently contain solo LTRs.

## RESULTS

### Study participants

Blood specimens were collected from pediatric study participants in the children with HIV early antiretroviral (CHER) study and post-CHER cohorts. The children’s viremia was suppressed for 7–9 years. HIV proviral integration sites were previously identified in samples taken before and on long-term cART (32). We investigated the structures of the proviruses in the nine largest infected cell clones from five of the children (Table 1). The children are referenced by the identifiers used in Bale, Katusiime et al. (32): ZA-004, ZA-005, ZA-007, ZA-010, ZA-011.

**TABLE 1:** The nine largest clones from five HIV-1-positive children initiated on cART within 10 mo of birth^*a*^

Donor	Age at cART initiation (months)	Sampling period after cART initiation (years)	Integrant chromosome/orientation	Gene/orientation	% of total integrations detected
ZA-007	9.9	8.1–9.6	C1/+	SRSF10/−	5.8%
			C1/−	TTC13/−	1.3%
			C11/−	RAB6A/−	1.9%
ZA-011	9.3	7.8–9.3	C2/−	ALMS1/+	1%
			C6/−	RANBP9/−	1.3%
ZA-004	2.7	9.2–10.7	C14/−	RAD51B/+	4.4%
			C6/−	Intergenic	2.2%
ZA-005	6.1	8.5–10.2	C6/−	Intergenic	6.4%
ZA-010	1.8	8.8–10.5	C6/−	Intergenic	12.5%

^*a*^ The table shows the donors studied and sampling periods analyzed. The characteristics of the integration site of each provirus are indicated, along with the size of the clone relative to other known proviruses.

### Integration site-specific proviral amplification

**Fig 1:**
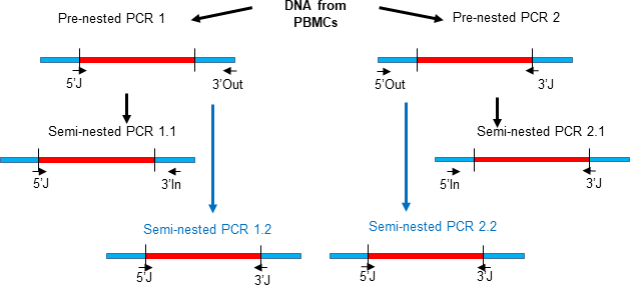
Integration site-specific amplification strategy. Human genomic sequences are shown in blue and proviruses in red. The PCR approach to identifing solo LTRs by pre-nested and semi-nested PCR reactions is shown schematically. The location and orientation of the first (Out) and second (In) PCR primers in the human genome and the integration site-specific junction (J) are indicated by horizontal arrows.

**Fig 2:**
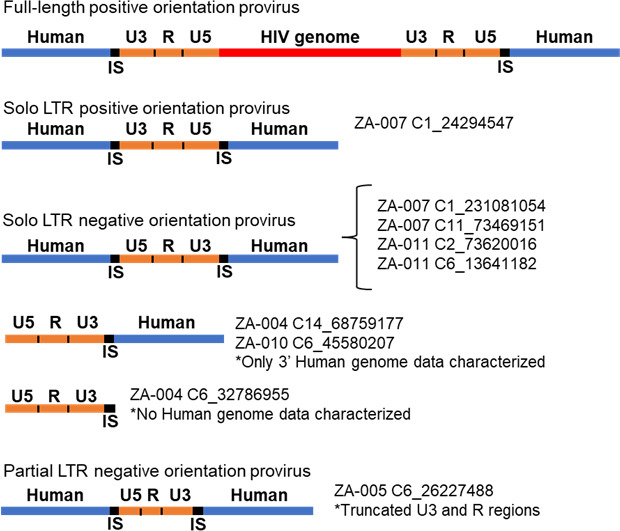
Proviral structure/orientation, integration site location, and human genome information of cell clones containing a solo LTR. A full-length provirus is included in the schematic for comparison. Clone integration sites (IS) are indicated along with the respective human chromosome sequences for each donor.

The integration sites of the proviruses in the nine largest clones had previously been characterized by integration-specific site assay (ISA), but the sequences of the proviruses were not known (9, 20) (Fig. 1). In brief (details in methods), primers were designed (Table S3) to amplify the full-length proviruses using four parallel semi-nested PCRs. The primers were designed based on the host-proviral junctions. DNA from peripheral blood mononuclear cells (PBMCs) was extracted, and the PCR was initiated using two pairs of primers. In each amplification, one primer spanned the host-virus junction and the other matched the host genome as shown in Fig. 1 (pre-nested 1 and 2, followed by semi-nested 1.1 and 2.1). A second amplification was done on pre-nested 1 and 2 products in which both pairs of primers spanned the host-virus junctions (Fig. 1, semi-nested 1.2 and 2.2). Unexpectedly, we found that all nine (seven confirmed and two probable) of the largest infected cell clones in the children contained proviruses that consisted of a single LTR (solo LTR) or a partial sequence of a single LTR (partial LTR) (Fig. 2).

Proviral sequences were obtained from multiple independent amplifications for each of the proviruses. LTR recombination can occur during PCR due to the matching LTR sequences at both ends of a provirus. For each of the nine proviruses, we attempted to amplify internal HIV sequences by priming from the host-virus junction to the HIV *gag* leader or downstream *gag* regions, but yielded no specific amplification products, as expected for solo LTRs. DNA from ACH2 cells was run in parallel as a positive control for full-length proviral amplification. Serially diluted ACH2 DNA amplified using the same reaction conditions that produced solo-LTR products from clinical samples yielded only ~9 kb (full-length provirus) products and no solo-LTR PCR recombination artifacts when the assays were run with low copy DNA input.

To further ensure that the detection of solo LTRs was not the result of PCR recombination, we used another PCR protocol that included the separation of proviral sequences in droplets on a subset of the clones that we reported to contain solo-LTR proviruses in the children. Further, we compared the PCR products from unsheared and sheared genomic DNA as starting materials. Our results confirmed that the proviral sequences in the children consisted of solo LTRs without signs of longer proviral sequences (Fig. S2, lanes 1–3). DNA shearing did not impair the amplification of the solo-LTR products (Fig. S2, lanes 4–6) as it did for a full-length provirus that was used as a control.

We also performed our PCR amplification approach on a sample with known intact and defective proviruses from an adult donor PID F07 (15) to further ensure that the detection of solo LTRs was not due to artifactual PCR recombination. We analyzed four infected cell clones in PID F07: two with solo LTRs, one with a full-length provirus, and one with a provirus with a 7 kb deletion spanning the end of *gag* to the R domain of the 3′-LTR (Fig. S3). Using our PCR amplification approach, we found no evidence of PCR recombination of the full-length or partially deleted provirus to generate artifactual solo LTRs.

Most of the semi-nested PCR reactions performed on the samples from the children were successful; however, there were some exceptions: the provirus at integration site “Chr14_RAD51B” in ZA-004 could only be amplified using PCR 2.1 and 2.2, and the provirus at site “Chr6_Intergenic” in ZA-010 could only be amplified using PCR 1.1 and 1.2 (Table S1). Amplification of provirus “Chr6_Intergenic” in ZA-004 was only successful after semi-nested reaction 1.2, yielding an LTR sequence without the human-provirus junction. We found no obvious link between the size of a clone and the successful amplification of the provirus.

### Phylogenetic analysis of the solo LTRs

Phylogenetic analysis shows that solo-LTR sequences from each child clustered within the HIV sequences found for that child and not with sequences from other children (Fig. S1). Four different amplification reactions, targeting each provirus from either side of the integration site (Fig. 1), yielded, for seven targeted proviruses, an identical solo-LTR proviral sequence and the corresponding integration site.

### Sizes of the infected cell clones

**Fig 3:**
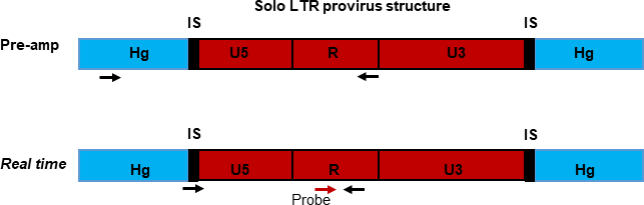
Representation of the integration site-specific proviral quantification method. This consists of a pre-amplification, a *real-time* amplification, and a quantification step. The section of the human genome (Hg) is indicated along with the integration site (IS) and proviral LTR. Primers are indicated by black horizontal arrows, and the probe is represented by a red horizontal arrow.

**Fig 4:**
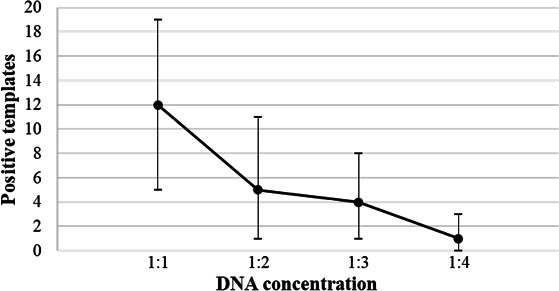
Error plot of provirus quantification by integration site quantification on serially diluted genomic DNA. A semi-linear dilution effect was observed when quantification was performed on serially diluted genomic DNA from patient ZA-007, targeting the provirus at Chr1_SRSF10. The expected number of positive templates is indicated by black dots, with the 95% confidence intervals of the values obtained by integration site quantification indicated by vertical lines.

**Fig 5:**
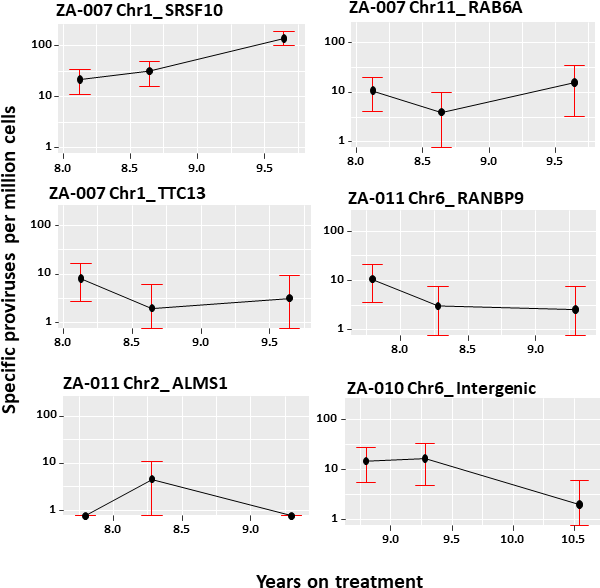
Changes in the sizes of clones containing solo LTRs over time indicated by years of cART. The numbers of specific proviruses were normalized per one million cell equivalents of genomic PBMC DNA, with error bars showing 95% confidence intervals (CI). The first clone (ZA-007 Chr1_SRSF10) showed a significant expansion in the normalized clone size between the second and third time points, as evident from non-overlapping CIs (*P* < 0.001). The last clone (ZA-010 Chr6_Intergenic) showed a significant decline between the first and last time points (Fisher exact *P* = 0.03). The data for the other clones did not show a significant difference in size during the sampling interval.

The proviral amplification assay was modified to a semi-nested real-time PCR, referred to as integration site-specific proviral absolute quantification (Fig. 3), so that it could be used to determine the sizes of the cell clones with solo LTRs at three timepoints over a 1.5-year period of sampling on virologically suppressive cART. No amplification was observed in patient-specific reactions spiked with ACH2 DNA, confirming assay specificity. The sizes of the clones were measured based on the fraction of wells that were positive when defined amounts of donor DNA were added to an integration site-specific PCR reaction. The effect of diluting the DNA was done with the provirus in Chr1_SRSF10 from donor ZA-007 to further validate the assay (Fig. 4). Changes in the sizes of the clones were determined when longitudinal samples were available, and amplification was successful for *N* = 6 clones (Fig. 5). The range of expected positive wells for each set of integration site-specific reactions (calculated by Poisson probability and 95% CI determined by non-parametric bootstrapping) was normalized using CCR5 quantification data (Table S1). The normalized data (Table S1) was used to construct line graphs with error bars 95% CI at each point (Fig. 5), showing the changes in the sizes of the clones over time (33). There was a significant change in the sizes of two of the clones: in donor ZA-007, clone Chr1_SRSF10 grew larger, and in ZA-010, clone Chr6_Intergenic decreased in size (Fisher exact *P* = 0.03).

### Integrase DNA copy number vs LTR copy number

**Fig 6:**
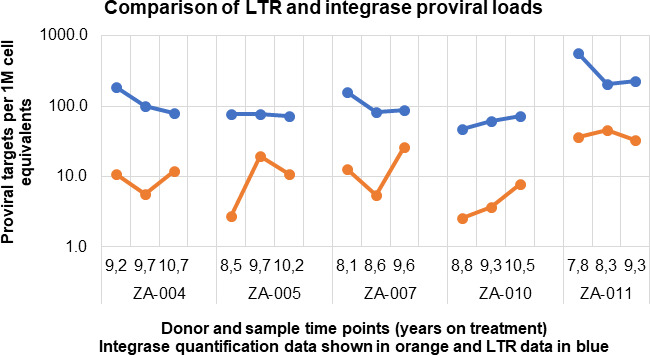
A comparison of the numbers of copies of LTR and integrase DNA over time. Log scale plot comparing the numbers of copies of integrase (orange) (determined by iCAD) and LTR (determined by LCAD) at three time points over ~1.5 years sampling period.

Integrase DNA copy number was determined by the integrase cell-associated DNA (iCAD) assay (34), adapted for subtype C (35), and compared to the LTR copy number. The integrase DNA to LTR ratio ranged from 1:3.3 to 1:28.6 across the different samples and donors (Fig. 6), consistent with a subset of the large infected cell clones containing solo-LTR proviruses.

## DISCUSSION

Although a profile of the deletions that are found in HIV proviruses has been presented (24, 25) and there has been additional characterization of both intact and defective proviruses at specific integration sites (15, 21, 22), the assays used in these reports would not have detected solo LTRs because they rely on priming of internal HIV sequence between LTRs. Anderson et al. (31) reported that a very large clone of HIV-infected cells (25% of the infected cells in the blood) in an adult carried a solo LTR (31) and that, in most other adults, the ratio of LTR sequences to internal HIV genes increased over time on ART, suggesting enrichment of proviruses consisting of solo LTRs. However, the structure of most of the proviruses was not determined, leaving the question open.

Solo-LTR proviruses are well described for a number of endogenous retroviruses (28, 30), including human endogenous retroviruses (HERVs) (29, 36, 37). Solo LTRs comprise as much as 90% of the HERVs in the human genome (38). However, the endogenous solo LTRs that arose by homologous recombination between the LTRs of endogenous proviruses accumulated over millennia, and it was not clear that solo LTRs would arise in PLWH.

The provirus in the largest infected cell clone in five children born with HIV and treated with cART for more than 6 years all contained solo LTRs, with one LTR having a 240-bp deletion in the R region. Although we do not have direct evidence, it is most likely that the solo HIV LTRs in the children were generated by recombination between the LTRs after the proviruses were integrated, as occurs in all known retroviruses. The generalizability of our findings is supported by Anderson et al., who describe LTR:*gag* ratios in adults of more than 10, suggesting that proviruses with large internal deletions (such as solo LTRs) accumulate over time on ART (31). Similarly, the high proportion of solo LTRs found in the current study provides insight into previous reports observing up to 10-fold higher HIV DNA quantities when targeting LTR compared with internal non-LTR proviral sequences (39).

To ensure that the solo-LTR proviruses that we detected were not the result of PCR recombination, we performed several control experiments. For example, we amplified a subset of the proviral sequences by emulsion PCR so that the individual proviruses remained separated in droplets. Physical separation of the proviruses prevented the formation of hetero-hybrids between proviruses from unrelated loci. Off-target priming events were also minimized by preventing primer trimming and by annealing the primers at stringently high temperatures. Under these stringent reaction conditions, the proviral sequences that were amplified in the designated T cell clones in the children consisted exclusively of solo-LTR products, and we did not observe longer amplified proviral sequences. Additionally, DNA shearing did not impair the amplification of solo-LTR products but did impair the amplification of an intact provirus that was used as a control for our approach. The lack of amplified long proviral sequences from intact DNA templates and the efficient amplification of solo-LTR sequences from sheared DNA templates argue strongly against possible artifacts due to PCR recombination-driven generation of solo LTR. Thus, the results obtained by our stringent PCR protocol confirmed our findings that the largest infected T cell clones identified contained solo-LTR proviral sequences.

The use of proviral amplification and quantification assays targets single-infected cell clones. The ability of the assay to detect differences in the size of clones depends on the number of cells assayed. Here, the size of the cell clones we analyzed was similar to the size of the same clones reported in Bale, Katusiime et al. (32), demonstrating the accuracy of our methods. Our findings are also supported by data from Bale, Katusiime et al. (32), which describe a 10-fold higher integration site detection compared to HIV *pol* copies measured in the children reported here (Table S2).

The preferential retention of solo LTRs may result from (1) their failure to produce viral proteins to be targeted by immune cells and (2) their increasing frequency from further post-integration homologous recombination of LTRs in intact and defective proviruses. The survival of the cells that carry solo LTRs (and other forms of highly deleted proviruses) and expand into predominant cell clones is supported by reports indicating that cells expressing HIV mRNA and protein are more likely to be purged by the immune system (40, 41). Although the large clones we characterized here contain solo LTRs, we do not imply that all large clones will contain solo LTRs, as it is well known that some large, infected T cell clones can carry proviruses with internal sequences and with intact proviruses (11).

Our findings that at least some of the largest infected cell clones in children on long-term cART contain solo LTRs pose several questions: (1) Are solo-LTR proviruses more prevalent in children than adults? (2) How rapidly do solo LTRs increase with time on cART (resulting from homologous recombination among LTRs in intact and/or defective proviruses)? and (3) Is the observed decrease in intact proviruses with time on cART reported by others (31, 42, 43) due to post-integration homologous recombination of LTRs (resulting in solo LTRs) and subsequent outgrowth of the clones that harbor them?

The HIV LTR carries a promoter that could regulate the expression or suppression of adjacent human genes (36, 44, 45). In adults, integrations in certain portions of seven genes (46) have been shown to confer selective advantages for cell survival and expansion. The solo-LTR proviruses we describe are not integrated in any of these seven genes, and there is no reason to suspect that the solo-LTRs we describe here contributed to the growth and survival of the clones. It would be important to understand whether any of the proviruses integrated in the appropriate regions of those seven genes consist of solo LTRs. However, although a solo HIV LTR contains *cis*-activating elements such as NF-kappaB-binding sites, it lacks other components that should be expected to help the expression of part or all of a host gene (the major splice donor, Tat, and, perhaps, in some cases, Rev) (47). Further investigation is required to determine the effects, if any, that solo LTRs have on adjacent host genes (48, 49). Additionally, observations of variability in clone size in this study, with two cases of definitive waxing and waning, may be in keeping with reported antigen-driven and cytokine-induced proliferation as reported by others (12 - 14).

The finding that at least a subset of the largest infected cell clones in children on long-term cART contains solo LTRs suggests that there is considerable homologous recombination of LTRs after integration and that the decline of intact proviruses with time on cART may be due, at least in part, to the generation of solo LTRs. Our findings also suggest that a large fraction of defective proviruses may be missed by near full-length proviral sequencing using primer(s) that anneal to internal HIV sequences, resulting in an overestimation of the percent of intact proviruses that persist during ART. Understanding the naturally occurring mechanisms that result in the formation of solo-LTR proviruses could lead to new strategies that enhance solo-LTR formation from intact proviruses, thereby decreasing the persistence of HIV reservoirs.

## MATERIALS AND METHODS

### Clinical specimens

Participants in this study were from the CHER and post-CHER cohorts. The CHER study was conducted in children with HIV in South Africa, diagnosed between 4 and 6 weeks of life, and investigated for clinical and immunological outcomes (50, 51). The post-CHER cohort is a subset of participants retained from the CHER trial to study neurocognitive outcomes and HIV-1 reservoirs. Longitudinal whole blood samples were collected twice annually as part of the CHER and post-CHER studies. Previous investigations found no evidence of ongoing replication for 7–9 years in a subset of these children studied (52). Analysis of integration sites in these participants indicated that their HIV persists in clones of infected T cells that were present before cART was initiated (32). PBMCs from five children for whom integration site analysis had been performed were selected for this study (Table S2). All donors had their viremia suppressed for the sampling interval used in this study.

### PBMC and nucleic acid isolation

PBMCs were isolated from freshly collected whole blood specimens from selected CHER donors according to the hanc cross-network PBMC processing protocol (www.hanc.info/labs/labresources/procedures/Pages/pbmcSop.aspx). DNA was isolated from PBMCs as described by Bui et al. (53).

### Provirus amplification assay design and validation

We developed an assay to amplify specific proviruses based on the sequences at their integration sites. A combined semi-nested PCR strategy was used to amplify each provirus and the appended human genome sequences at both the 5′ and 3′ ends of the HIV LTRs (Fig. 1). The integration site-specific proviral amplification method consists of two parallel pre-nested reactions: reaction 1 primes from the human genome beyond the 3′ end of the provirus, with the opposing primer matching the HIV 5′ U3 LTR-human genome junction, while reaction 2 primes from the human genome beyond the 5′ end of the provirus, with the opposing primer matching the HIV 3′ U5 LTR-human genome junction. Each of these PCR reactions is followed by two semi-nested reactions, using the same junction primers as in the pre-nested reactions but opposing internal human genome primers, or alternatively, junction primers: reaction 1.1 and 1.2, and 2.1 and 2.2, respectively (Fig. 1). The four reactions amplify specific proviruses by targeting unique integration site junctions and human genome positions.

Amplification was performed with Ranger mix (Bioline meridian bioscience) and each reaction (50 µL) consisted of 1× Ranger mix, 0.4 µM forward and reverse primer, respectively, and nuclease-free water (Promega Corp.). Amplification conditions consisted of an initial denaturing step (95°C, 1 min) followed by 30 cycles of denaturation (98°C, 15 s), annealing (first five cycles consisted of touchdown temperature starting at 63°C down to 58°C, with the remaining 25 cycles at 58°C for 45 s), and extension (68°C, 11 min). A final extension step (68°C, 15 min) was included to ensure full-length products.

The proviral amplification assay was tested on ACH2 genomic DNA, targeting the intact proviral clone, to confirm that the assay conditions would amplify a full-length provirus. ACH2 DNA, serially diluted to five copies of the targeted provirus per reaction, was added to negative donor PBMC DNA (17,000 copies) to test the sensitivity of the assay. Due to the low level of the targeted integration sites, each proviral amplification assay (3′ and 5′) was performed for up to 33 replicates for each of the 9 selected integration sites until a positive amplicon was achieved. Amplicons of various sizes from ~600 bp to ~10 kb were Sanger sequenced.

### Emulsion PCR controls

Proviruses in clones were amplified by emulsion PCR in 20 µL reaction mixes with KAPA HiFi HotStart ReadyMix (2×) (Roche #07958935001), 100 ng of genomic DNA from PBMC, and 100 nM of each primer. The primers were designed with two phospho-thioester bonds at the 3′-termini (Table S4). Whenever specified, genomic DNA was sheared for 20 min in miniTUBE clear (Covaris #520064), DNA was repaired with NEBNext Ultra II Module (NEB #E7546) and cleaned with an equal volume of SPRIselect suspension (Beckman #B23318). Reaction mixes were emulsified in a BioRad droplet generator (BioRad #1864002). Proviral sequences were amplified by initial DNA denaturation for 3 min at 95°C, followed by 35 cycles of 30 s at 95°C, 30 s at 68°C, and 10 min at 72°C, and a final extension for 10 min at 72°C. Emulsions were broken with 100 µL of purification buffer (PB) (QiaQuick PCR purification kit, Qiagen #28106). PCR products were purified with 100 µL of SPRIselect suspension and eluted in 100 µL of elution buffer (EB). The second-round PCR was performed in 40 µL using 1 µL of the first-round PCR and proviral sequences were amplified following the protocol described above. PCR products were analyzed on Genomic Tapes (Agilent #5067–5365). Less than 1 kb PCR products were further analyzed by Sanger sequencing with each one of the PCR primers (Psomagen); >1 kb PCR products were analyzed by PacBio sequencing (Long Read Technology, Center for Cancer Research, NCI, Frederick, USA).

### Nucleotide sequencing and analyses

Selected PCR amplicons were gel purified using the Nucleotrap kit (Macherey-Nagel, Düren, Germany) according to the manufacturer’s instructions. Purified amplicons were Sanger sequenced with integration site-specific primers. Nucleotide sequences were verified and assembled using Sequencher 4.10.1 (Gene Codes Corp., Ann Arbor, MI, USA), Geneious Prime 2020.1 (Biomatters Ltd., Auckland, NZ) and BioEdit Sequence Alignment Editor (V7.2.5). Nucleotide sequences were identified as HIV by BLASTn (https://blast.ncbi.nlm.nih.gov/Blast.cgi?PROGRAM=blastn&PAGE_TYPE=BlastSearch& BLAST_SPEC = OGP__9606__9558&LINK_LOC = blasttab&LAST_PAGE = blastn). Proviral sequences were identified using sequences for the human genome that were specific to each integration site and confirmed by Human BLAT Search (https://genome.ucsc.edu/cgi-bin/hgBlat). Proviral sequences were reconstructed with sequence data obtained from the Sanger sequencing reactions and the closest HIV sequence match in GenBank (AF321523).

### Provirus quantification assay design and validation

A nested PCR approach was selected for sensitive and specific quantification of rare HIV proviruses. Increased sensitivity and specificity for the integration site-specific proviral absolute quantification assay is achieved by including a pre-amplification step before real-time quantification (Fig. 3), similar to assays described by (54). With the expected integration site-specific proviral load being less than one copy per reaction, multiple proviral amplification reactions were needed to detect a specific provirus, essentially using a “digitalized” 92 reaction Poisson quantification approach. Using this approach, PBMC DNA is dispersed at the endpoint into 92 reactions at a concentration of ~200 ng per reaction.

A first round (pre-amplification) step was performed with Ranger mix (Bioline meridian bioscience) with each reaction (20 µL consisting of 1× Ranger mix, 0.4 µM forward and reverse primer, respectively, and nuclease-free water (Promega Corp.). The pre-real-time conditions consisted of an initial denaturing step (95°C, 1 min) followed by 15 cycles of denaturation (98°C, 15 s), annealing (55°C, 15 s), and extension (68°C, 30 s). A final extension step (68°C, 5 min) was included to ensure a full-length amplicon. The real-time step was performed with LightCycler 480 Probes Master (Roche), and each reaction (25 µL) consisted of 1× LightCycler mix, 0.4 µM forward and reverse primer, respectively, 0.3 µM probe, nuclease-free water (Promega Corp.), and 5 µL pre-amplicon. Quantification conditions consisted of an initial denature step (95°C, 5 min) followed by 50 cycles of denaturation (95°C, 15 s), annealing (55°C for 15 s), extension (68°C, 30 s), and fluorescence measurement for FAM dye. Amplification curves were visually assessed, with a sigmoidal shape indicative of exponential amplification as a criterion for positivity, to determine the number of positive reactions per 92 integration site quantification reactions.

Poisson distribution statistics were used to determine the expected number of specific proviral templates per positive reaction. In order to accurately quantify specific infected cell clones, the number of cell equivalent DNA copies used for each integration site quantification run was determined. The highly sensitive CCR5 quantification assay described in Malnati et al. (55) was selected to determine the DNA input copy number. Determining the cell equivalent DNA copies used for each integration site quantification run allows for the normalization of quantification data to compare intra- and interpatient-specific clone quantification data longitudinally.

### Integrase DNA copy number vs LTR copy number

The iCAD PCR assay (34), adapted for HIV-1 subtype C (35), and an LTR CAD (LCAD) assay were performed on the three time points for all five donors. Results were normalized to HIV-1 DNA copies per 1 million cells, as per the CCR5 targeted assay described by Malnati et al. (55). LCAD is based on the same principle as iCAD and is performed in the same manner. The LCAD assay targets the HIV LTR in the R and U5 regions with primers 5′-GCC TCA ATA AAG CTT GCC-3′ and 5′-GGT CTG AGG GAT CTG TAG TTA-3′ at 0.4 µM each and probe 5’−56-FAM/AAG TAG TGT /ZEN/GTG CCC GTC TGT TGT /3IABkGQ-3′ at 0.3 µM.

## SUPPLEMENTAL MATERIAL

Supplemental Figure Legends mbio.01116-23-s0001.docx Legends for Fig. S1 to S3.

Figure S1 mbio.01116-23-s0002.tif Phylogenetic tree.

Figure S2 mbio.01116-23-s0003.tif Amplification of proviral sequences from PID ZA-007.

Figure S3 mbio.01116-23-s0004.tif Amplification of proviral sequences from four infected T cell clones in PID F07.

Table S1 mbio.01116-23-s0005.docx Integration site positive PCR reactions.

Table S2 mbio.01116-23-s0006.docx Integration site data from five donors.

Table S3 mbio.01116-23-s0007.docx Integration site-specific amplification primers.

Table S4 mbio.01116-23-s0008.docx Primers for confirming solo LTRs.

ASM does not own the copyrights to Supplemental Material that may be linked to, or accessed through, an article. The authors have granted ASM a non-exclusive, world-wide license to publish the Supplemental Material files. Please contact the corresponding author directly for reuse.
