# Supplementary material for: The largest HIV-1-infected T cell clones in children on long-term combination antiretroviral therapy contain solo LTRs
Source: mBio. 2023 Aug 2;14(4):e01116-23. doi: 10.1128/mbio.01116-23 (PMC10470503; doi:10.1128/mbio.01116-23)
Supplement: Supplemental Figure Legends — Legends for Fig. S1 to S3. [file mbio.01116-23-s0001.docx]

Supplementary Figure 1: Phylogenetic tree of HIV solo LTR proviral nucleotide sequences (634 bp).

Replicates of 1000 bootstraps were used with bootstrap percentage values indicated next to the branches. The closest HIV subtype C sequence match from GenBank was used as reference and root.

Supplementary Figure 2: Amplification of proviral sequences from PID ZA-007. The amplified proviruses are named after the gene in which they are integrated (SRSF10, RAB6A, TTC13). Genomic DNA template was either unsheared or sheared to 3-7 kb (mean 5 kb). The primers annealed to the host-LTR junctions as described in the methods. The primers for the nested PCR were shifted at least 6 nucleotides downstream. The PCR products were analyzed on a Genomic Tape Station (Agilent).

Supplementary Figure 3. Amplification of proviral sequences from 4 infected T cell clones in PID F07 (15). The amplified proviruses are named after the gene in which they are integrated (EVI2B, CLEC2B, NUP62, ATRX). The primers annealed to the host-LTR junctions as described in the meothods. The primers for the nested PCR were shifted at least 6 nucleotides downstream. The PCR products were analyzed on a Genomic Tape Station (Agilent).
