# Supplementary figures and images for: The largest HIV-1-infected T cell clones in children on long-term combination antiretroviral therapy contain solo LTRs
Source: mBio. 2023 Aug 2;14(4):e01116-23. doi: 10.1128/mbio.01116-23 (PMC10470503; doi:10.1128/mbio.01116-23)

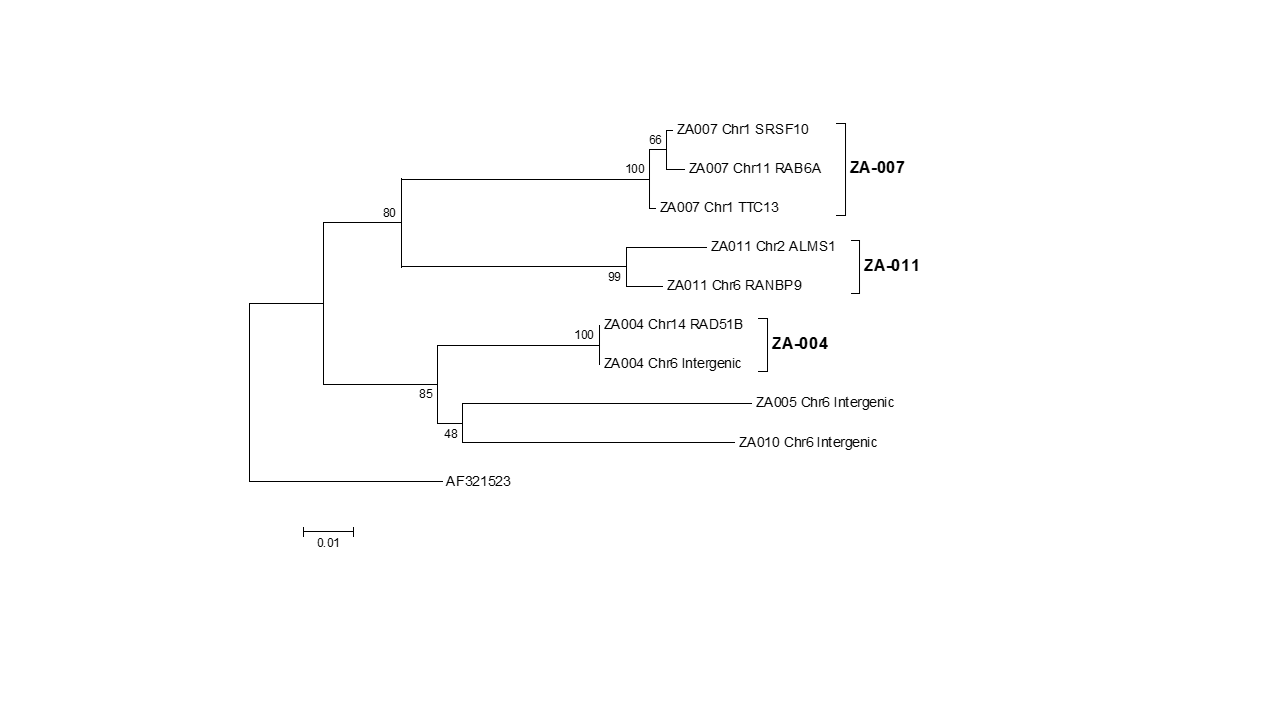

Supplement: Figure S1 — Phylogenetic tree. [file mbio.01116-23-s0002.tif]

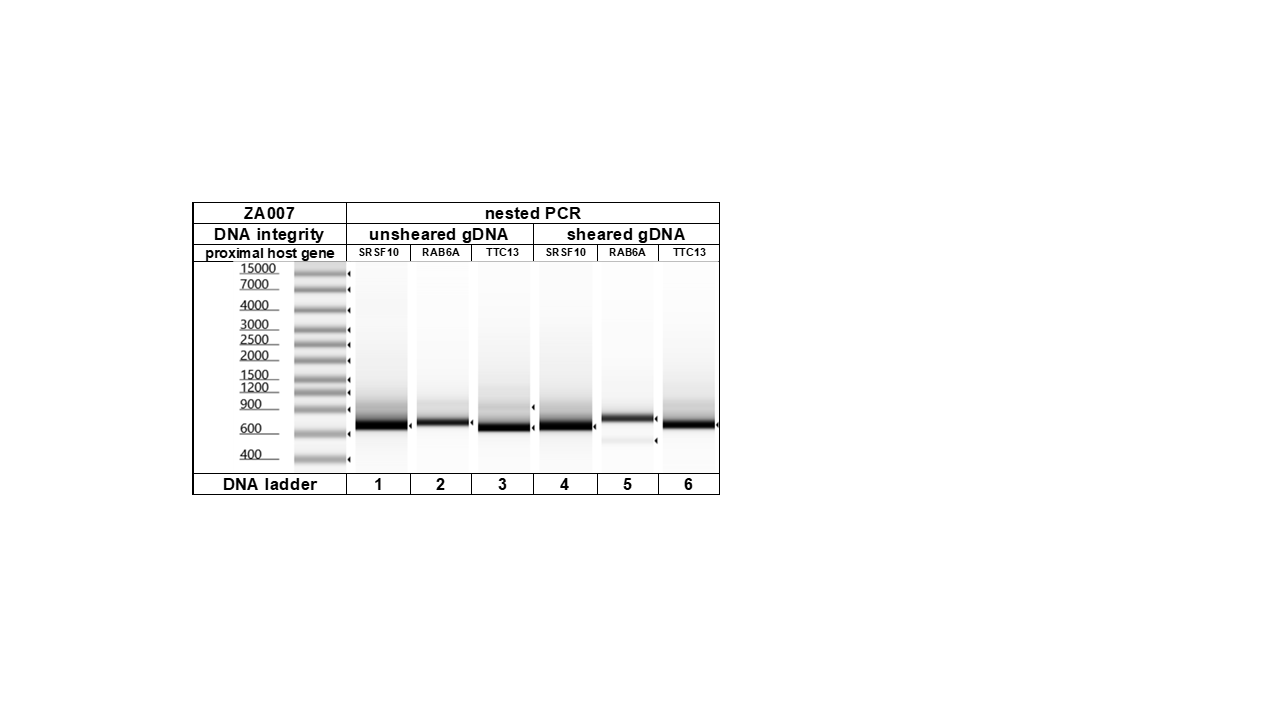

Supplement: Figure S2 — Amplification of proviral sequences from PID ZA-007. [file mbio.01116-23-s0003.tif]

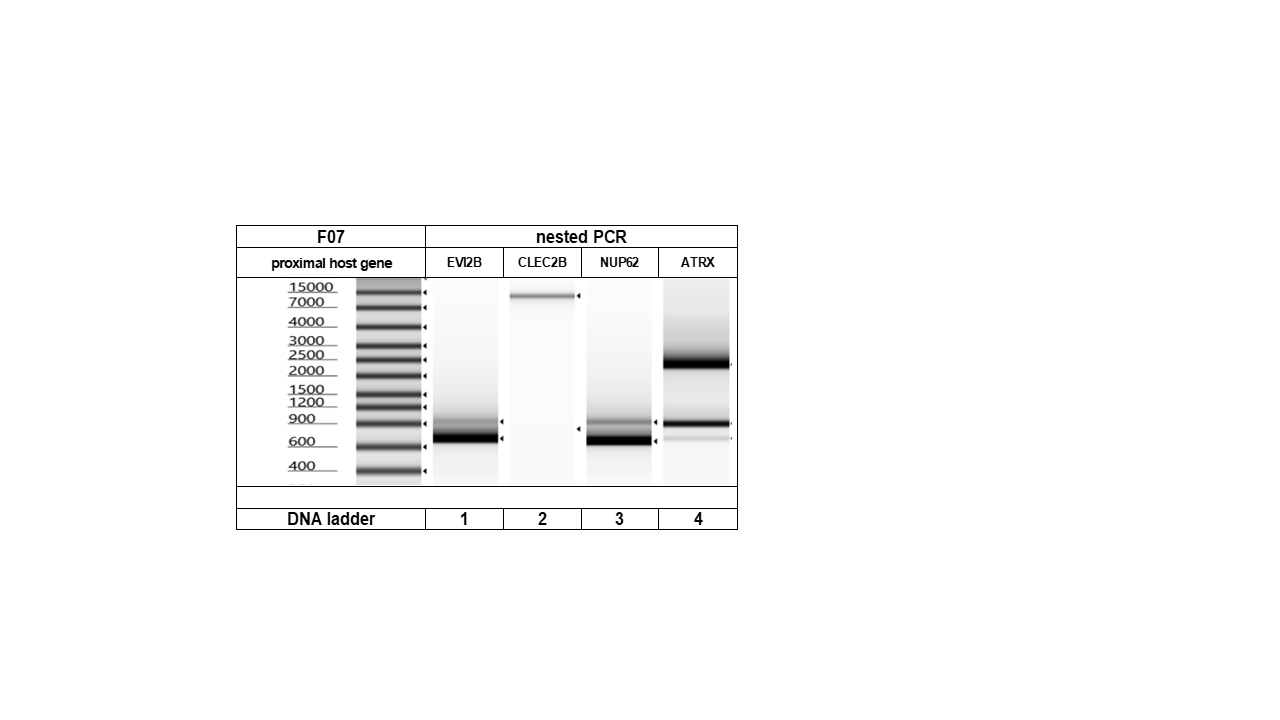

Supplement: Figure S3 — Amplification of proviral sequences from four infected T cell clones in PID F07. [file mbio.01116-23-s0004.tif]
