# Supplementary material for: The largest HIV-1-infected T cell clones in children on long-term combination antiretroviral therapy contain solo LTRs
Source: mBio. 2023 Aug 2;14(4):e01116-23. doi: 10.1128/mbio.01116-23 (PMC10470503; doi:10.1128/mbio.01116-23)
Supplement: Table S1 — Integration site positive PCR reactions. [file mbio.01116-23-s0005.docx]

Supplementary Table 1: Number of integration site specific positive PCR reactions for each of the clones at each time point. The range (low, expected (exp) and high) of positive reactions was calculated by Poisson and bootstrapping. The proviral quantification results were normalised to proviral copies per one million cell equivalent genomic DNA copies.

| Donor | Clone | Proviral copies per 1 million cell equivalent DNA copies | | | | | |
| --- | --- | --- | --- | --- | --- | --- | --- |
|  |  | **Time point 1** | | **Time point 2** | | **Time point 3** | |
|  |  | **Exp** | Low, High | **Exp** | Low, High | **Exp** | Low, High |
| ZA-007 | C1_SRSF10 | **22** | 11, 34 | **32** | 16, 50 | **140** | 98, 190 |
|  | C1_TTC13 | **11** | 4, 20 | **4** | 0, 10 | **16** | 3, 35 |
|  | C11_RAB6A | **8** | 3, 16 | **2** | 0, 6 | **3** | 0, 10 |
| ZA-011 | C2_ALMS1 | **11** | 4, 21 | **3** | 0, 8 | **3** | 0, 8 |
|  | C6_RANBP9 | **-** | - | **5** | 0, 11 | **-** | - |
| ZA-004 | C14_RAD51B | No quantification data obtained | | | | | |
|  | C6_Intergenic |  |  |  |  |  |  |
| ZA-010 | C6_Intergenic | **15** | 6, 28 | **17** | 5, 33 | **2** | 0, 6 |
