# Supplementary material for: The largest HIV-1-infected T cell clones in children on long-term combination antiretroviral therapy contain solo LTRs
Source: mBio. 2023 Aug 2;14(4):e01116-23. doi: 10.1128/mbio.01116-23 (PMC10470503; doi:10.1128/mbio.01116-23)
Supplement: Table S2 — Integration site data from five donors. [file mbio.01116-23-s0006.docx]

Supplementary Table 2: All integration site data obtained from the 5 donors. The total number of integration sites detected by the ISA assay is indicated along with the number of clones identified (32). The copy number of HIV integrase DNA (iCAD value) per 1 million PBMCs is included for reference.

| Donor | Age at treatment initiation (months) | Time to viral suppression (years) | Sampling period  after cART initiation (years) | iCAD value | Number of integration sites detected | Number of clones detected |
| --- | --- | --- | --- | --- | --- | --- |
| ZA-004 | 2.7 | 1.38 | 7.9 | 23.6 | 272 | 21 |
| ZA-005 | 6.1 | 0.47 | 8 | 9.2 | 94 | 3 |
| ZA-007 | 9.9 | 0.92 | 8.2 | 21.3 | 311 | 9 |
| ZA-010 | 1.8 | 0.46 | 8.4 | 4.5 | 128 | 3 |
| ZA-011 | 9.3 | 2.29 | 7.4 | 181.5 | 301 | 6 |
