# Supplementary material for: The largest HIV-1-infected T cell clones in children on long-term combination antiretroviral therapy contain solo LTRs
Source: mBio. 2023 Aug 2;14(4):e01116-23. doi: 10.1128/mbio.01116-23 (PMC10470503; doi:10.1128/mbio.01116-23)
Supplement: Table S3 — Integration site-specific amplification primers. [file mbio.01116-23-s0007.docx]

Supplementary Table 3: Primers used for integration site specific proviral amplification. Primer names indicate targets based on human chromosome and location of integration site.

| Primer name | Sequence |
| --- | --- |
| C14_68759177_5Out | GTT GGC TTT CCC AGT GGT CC |
| C14_68759177_5In | TGC TGA GTT CTT TTG GAC TAA AAA GCG |
| C14_68759177_5J | GTA AAT CTG CAA AAT TTT CTC CAC TGC TAG |
| C14_68759177_3J | GGG AGG GTG GAT GGA AGG G |
| C14_68759177_3In | CCA GAC CCA CAG CAT TCT GC |
| C14_68759177_3Out | TGG AAG AGC TAT GCA TGT CTA CCC |
| C6_32786955_5Out | CAT CAT CTT TCA GCA TGA AAT GTG CC |
| C6_32786955_5In | TGA AAT GTG CCC CTG ATT TGC C |
| C6_32786955_5J | TCC AGG GCC ATG ACT GCT AG |
| C6_32786955_3J | GCT AGA AGT TCA AGG TCA TTG GAA GG |
| C6_32786955_3In | AGC CTT ATG CTC TCT GTC TTA GTC C |
| C6_32786955_3Out | TCT GAA GAA GTG TAC CCT TCT TCC C |
| C6_26227488_5Out | GGT CTT GTG AAT TGG AGA TTC AGT GC |
| C6_26227488_5In | GCG TGA GGA AAA CAG CCT AAC TAT CC |
| C6_26227488_5J | AAT GTT GTT TCC TGT ATC CTG TAT GCT AG |
| C6_26227488_3J | GCT AGA ATC TTA AAG AAA GTA CAG TGG AAG G |
| C6_26227488_3In | AAA AGA TGC ATT CTG TGC CCA CC |
| C6_26227488_3Out | AGA GGC TTT GTT TTA GGC AAA TGA CC |
| C1_24294547_5Out | TCT CTC ACT GAA GCA TTA TCT CTC |
| C1_24294547_5In | TGT GTC AGC CCT CAT TTG AGT G |
| C1_24294547_5J | ACC ATT CTA TTT ATC ATT GAG CTG GAA G |
| C1_24294547_3J | GTA TGA CTG GGC TCA TGC TAG AG |
| C1_24294547_3In | GAT CCT ACT GAT ACT TGA TGA GG |
| C1_24294547_3Out | ACA CCT AAC AGC TTC AAA AGG AC |
| C11_73469151_5Out | GTT GCA TTC TTC TGT GGG |
| C11_73469151_5In | CTT TGA AAG CTG AAT TGT GG |
| C11_73469151_5J | CAT GAA GAG TTA CTG CTA G |
| C11_73469151_3J | CTT TTG ATG TAA CTG GAA GG |
| C11_73469151_3In | TGT CTA TAG TTT TCT TAT GCG |
| C11_73469151_3Out | ACT ATA TTG TCA AGT AAT GCC |
| C1_231081054_5Out | GAT CTG GGT GGA AAT CC |
| C1_231081054_5In | TTT CTC ATC TGT GAA CTG G |
| C1_231081054_5J | GGT CAA TGA ACA GTG CTA G |
| C1_231081054_3J | AAC TGC TAC TGT TTG GAA G |
| C1_231081054_3In | GTT ACG ATT GCC ATA GCG |
| C1_231081054_3Out | GGT TAG TGC AAT TTA TGG C |
| C6_45580207_5Out | TAT TGA AAG CTC CCC AAG GAG AGG |
| C6_45580207_5In | CCA AGG AGA GGG GCT ATG C |
| C6_45580207_5J | TGG TCC TCC TTG ATT CCT GCT AG |
| C6_45580207_3J | TAG GGA TGA AGG AGG AAT TGG ATG G |
| C6_45580207_3In | CCA GTG GAA TAA TAC TCA GCA GTA AAG AGG |
| C6_45580207_3Out | AGG TGT CAA ATA CCT GTC CAG TGG |
| C2_73620016_5Out | CTA TTA TGT ATA ATG CTA CCG |
| C2_73620016_5In | GGA TTC ATT TCT CTT GGA TG |
| C2_73620016_5J | TTT TGG GAC ATA TGT GCT AG |
| C2_73620016_3J | CAG AAT TCC ATA TTG GAA GG |
| C2_73620016_3In | GCC AGA TTG TTT TCC AAA G |
| C2_73620016_3Out | GGA ACT CTC ATA CAC TGC |
| C6_13641182_5Out | CAA AAT GTA GAA TGG TGG TTT CCA GGG |
| C6_13641182_5In | GGA GTC GGA TGG TGG TGA TGG |
| C6_13641182_5J | TAG ACA CCC ATC TTC TAA ATA AGT GCT AG |
| C6_13641182_3J | GTT TAA AAA TGT GAA AAT CTT ATT GGA TGG G |
| C6_13641182_3In | GCA GGA AGA ATG GGA GAA GCC |
| C6_13641182_3Out | CAG AAA TTG GTA TTA GCA GGA AGA ATG GG |
