# Supplementary material for: The largest HIV-1-infected T cell clones in children on long-term combination antiretroviral therapy contain solo LTRs
Source: mBio. 2023 Aug 2;14(4):e01116-23. doi: 10.1128/mbio.01116-23 (PMC10470503; doi:10.1128/mbio.01116-23)
Supplement: Table S4 — Primers for confirming solo LTRs. [file mbio.01116-23-s0008.docx]

Supplementary Table 4: Primers for amplification of proviral clones from F07 (15) and ZA-007 (this study). The primers annealed to the host-LTR junctions. The 3’-termini of the primers for the first-round PCR matched four nucleotides to the LTR. The primers for the second-round PCR were shifted at least six nucleotides downstream. The primers were designed with melting temperature of 68ºC using the T_m_ calculator of NEB for Q5 polymerase.

| Primer | Sequence |
| --- | --- |
| EVI-5J1 | CTTCCTTATAAAGTCATTTGAAGGTTCTGTGAAATTGGA |
| EVI-5J2 | TGAAGGTTCTGTGAAATTGGAAGGGCTAG |
| EVI-3J1 | TGTTGTACTATCCCAGTTTTGCATACTTTATTTCTGCT |
| EVI-3J2 | CCAGTTTTGCATACTTTATTTCTGCTAGAGATTTTCCAC |
| CLEC-5J1 | CCTAACATGACAAGACATAAATTTTTTTCAACCTGTTATATGATATTGGA |
| CLEC-5J2 | GACATAAATTTTTTTCAACCTGTTATATGATATTGGAAGGGCTAG |
| CLEC-3J1 | GATGTAAAAGATTATGGTCACAGCAAAATTTGTATATCTGCT |
| CLEC-3J2 | GGTCACAGCAAAATTTGTATATCTGCTAGAGATTTTCC |
| NUP-5J1 | CCTTAAAATCTCTCTCTGGCTGTTTCCTAATGGA |
| NUP-5J2 | TCTCTCTGGCTGTTTCCTAATGGAAGG |
| NUP-3J1 | GCTAAGACAACCATGGAAAAGTTAGGTGCT |
| NUP-3J2 | ACCATGGAAAAGTTAGGTGCTAGAGATTTTCC |
| ATRX-5J1 | TGTAATTTTTGTGAACCTTAAACTGTTTTTGAAAGGTGGA |
| ATRX-5J2 | GTGAACCTTAAACTGTTTTTGAAAGGTGGAAGG |
| ATRX-3J1 | TGACAACTCATGGTACTATCTTTTTGTTTTTATTATAACCTTTTGCT |
| ATRX-3J2 | TCATGGTACTATCTTTTTGTTTTTATTATAACCTTTTGCTAGAGATTTTCC |
| SRS-5J1 | CATTTGAGTGTTGATATAACCATTCTATTTATCATTGAGCTGGA |
| SRS-5J2 | CCATTCTATTTATCATTGAGCTGGAAGGGTTAATTTACTC |
| SRS-3J1 | CTACTGTGTATGACTGGGCTCATGCT |
| SRS-3J2 | CTGGGCTCATGCTAGAGATTTTCCAC |
| RAB-5J1 | TGTAGACTCCATGTCCTTTTGATGTAACTGGA |
| RAB-5J2 | TGTCCTTTTGATGTAACTGGAAGGGTTAATTTACTCT |
| RAB-3J1 | GGAATTCTAACAGACCTAAAAATAAATAATTCATGAAGAGTTACTGCT |
| RAB-3J2 | CTAAAAATAAATAATTCATGAAGAGTTACTGCTAGAGATTTTCCACACTAC |
| TTC-5J1 | GGGTTTTGCTAGAACTGCTACTGTTTGGA |
| TTC-5J2 | AGAACTGCTACTGTTTGGAAGGGTTAATTTACTC |
| TTC-3J1 | CCCATCAAGGGTCAATGAACAGTGCT |
| TTC-3J2 | GGGTCAATGAACAGTGCTAGAGATTTTCCTC |
